# Tumour-derived exosomes and their role in cancer-associated T-cell signalling defects

**DOI:** 10.1038/sj.bjc.6602316

**Published:** 2005-01-18

**Authors:** D D Taylor, C Gerçel-Taylor

**Affiliations:** 1 Department of Obstetrics, Gynecology & Women's Health, University of Louisville School of Medicine, Louisville, KY 40202, USA; 2 Department of Radiation Oncology, University of Louisville School of Medicine, Louisville, KY 40202, USA

**Keywords:** exosomes, activation signaling, membrane vesicles, T lymphocytes, ovarian cancer

## Abstract

Dendritic and lymphoid ‘exosomes’ regulate immune activation. Tumours release membranous material mimicking these ‘exosomes,’ resulting in deletion of reactive lymphocytes. Tumour-derived ‘exosomes’ have recently been explored as vaccines, without analysis of their immunologic consequences. This investigation examines the composition of tumour-derived ‘exosomes’ and their effects on T lymphocytes. Membranous materials were isolated from ascites of ovarian cancer patients (*n*=6) and Western immunoblotting was performed for markers associated with ‘exosomes.’ Using cultured T cells, ‘exosomes’ were evaluated for suppression of CD3-*ζ* and JAK 3 expressions and induction of apoptosis, measured by DNA fragmentation. ‘Exosome’ components mediating suppression of CD3-*ζ* were isolated by continuous eluting electrophoresis and examined by Western immunoblotting. ‘Exosomes’ were shown to be identical with previously characterised shed membrane vesicles by protein staining and TSG101 expression. ‘Exosomes’ expressed class I MHC, placental alkaline phosphatase, B23/nucleophosmin, and FasL. ‘Exosomes’ suppressed expression of T-cell activation signalling components, CD3-*ζ* and JAK 3 and induced apoptosis. CD3-*ζ* suppression was mediated by two components: 26 and 42 kDa. Only the 42 kDa component reacted with anti-FasL antibody. These results indicate that, while ‘exosomes’ express tumour antigens, leading to their proposed utility as tumour vaccines, they also can suppress T-cell signalling molecules and induce apoptosis.

One general characteristic of tumours is their ability to release or shed intact, vesicular portions of membrane material (termed membrane fragments, membrane vesicles, microvesicles or exosomes), which was initially described by us, 25 years ago (Taylor and Doellgast, 1979; Taylor *et al*, 1980). Recently, these tumour-derived vesicles, or ‘exosomes,’ have gained attention as a source of tumour antigens for vaccines, either directly or as part of processed dendritic cells or dendritic cell-derived vesicles (André *et al*, 2002a, 2002b; Chaput *et al*, 2003; Kim *et al*, 2004).

Membrane vesicle shedding and its accumulation appear to be important features of the malignant transformation. Shed tumour-derived membrane vesicles do not mirror the general composition of the plasma membrane of the originating tumour cell, but represent ‘micromaps’, with enhanced expression of tumour antigens (Taylor and Black, 1986, 1987; Taylor *et al*, 1988). While the precise mechanism of shedding remains unclear, the release of membrane vesicles has been demonstrated to be an energy-requiring phenomenon of viable cells, modulated by extracellular signals (Taylor *et al*, 1983a, 1988). The rate of shedding is significantly increased in most neoplastic cells and occurs continuously (Taylor and Black, 1986, 1987; Taylor *et al*, 1983a, 1988, 2002). In addition to cancer cells, the release of membrane vesicles was also demonstrated to be associated with cells of embryonic origin (such as the placenta) and activated lymphoid cells (Taylor and Black, 1986, 1987; Heijnen *et al*, 1999; Denzer *et al*, 2000). Although extracellular shedding of membrane-derived vesicles occurs in other types of cells, under specific physiological conditions, the accumulation of shed membrane vesicles from non-neoplastic cells is rarely observed (Taylor and Black, 1986; Taylor *et al*, 2002).

Since shed membrane vesicles exhibit molecules with biologic activity (such as Fas ligand, mdr 1, CD44, and autoreactive tumour antigens) (Taylor *et al*, 1983b, 1984, 1989, 1996, 2002), the ability of these shed membrane vesicles to modulate lymphocyte and monocyte functions have been analysed in several tumour models. Membrane vesicles released by metastatic tumour cells suppressed the expression of class II MHC antigens by monocyte/macrophages in a dose-dependent manner, while similar vesicles from early stage tumour cells did not (Taylor and Black, 1985; Poutsiaka *et al*, 1985a, 1985b). The shed membrane vesicles were also shown to suppress lymphocyte activation with phytohaemoglutinin, Concanavalin A, or antiCD3 (Taylor *et al*, 1988). These shed tumour-derived membrane vesicles also inhibited the activation of lymphocytes with interleukin-2 (Pelton *et al*, 1991). Our recent findings have demonstrated that these membrane vesicles can also suppress the expression of CD3-*ζ* and subsequent activation signalling, inhibiting proliferation and cytokine production (Taylor *et al*, 2003).

We have theorised that these shed vesicles modulate lymphocyte functions by mimicking ‘activation-induced cell death’ (AICD) (Taylor and Black 1986; Martinez-Lorenzo *et al*, 1999). Lymphoid cells appear to release membrane vesicles following activation and these appear to play an essential role in immunoregulation, preventing of excessive immune responses and the development of autoimmunity (Green *et al*, 2003). It was postulated that membrane shedding by tumour cells was a re-expression of the fetal cell shedding and that both constituted pathways to circumvent immunosurveillance (Gerçel-Taylor *et al*, 2002).

These shed membrane vesicles were rediscovered in the mid-1980s and termed ‘exosomes’ (Pan *et al*, 1985; Johnstone *et al*, 1987). These ‘exosomes’ were postulated to be functional extensions of antigen-presenting cells, stimulating immune responses (Théry *et al*, 2002). Théry characterized the composition and physical properties of this membranous material, demonstrating the ability of the centrifugal isolation procedure differentiated between exosomes and other vesicular structures and large protein aggregates Théry *et al* (1999). While characterizations of ‘exosomes’ have been consistent with our previously described shed tumour-derived membrane vesicles, in terms of size (60–100 nm in diameter) and general composition, the term, ‘exosomes,’ has separated its literature from the published immunologic activities of tumour-derived membrane vesicles. In the present study, we are attempting to bridge this gap, to definitively show identity between ‘exosomes’ and membrane vesicles and to establish that these shed ‘exosomes’ possess immunosuppressive activity.

## MATERIALS AND METHODS

### Patient-derived materials

Ascites were obtained from women diagnosed with stage IIIc papillary serous adenocarcinoma of the ovary (*n*=6) in the Gynecologic Oncology Clinic of the Department of Obstetrics and Gynecology of the University of Louisville School of Medicine. Blood samples were also obtained from normal female volunteers (*n*=8) from the Gynecology Clinic of the Department of Obstetrics and Gynecology of the University of Louisville School of Medicine. The University Human Studies Committee of the University of Louisville approved this study and informed consent was obtained from each patient. Blood samples were allowed to clot and then were centrifuged at 400 **g** for 10 min to sediment cells and clot. The serum was removed, aliquoted, and stored at −70°C until analysis. For all of the samples studied, the age of the nontumour-bearing female volunteers was 57.2±4.1 years, compared to 59.4±5.3 years for women with ovarian cancer.

### Isolation of tumour-derived membrane vesicles

Membrane vesicles were isolated from ascites by two separate procedures and then compared. The first protocol was the two-step chromatography/centrifugation procedure developed in our laboratory (Taylor and Doellgast, 1979; Taylor *et al*, 2002) and the second was the density gradient centrifugation procedure described for ‘exosome’ isolation (Raposo *et al*, 1996). In the first chromatography procedure, 10 ml of ascites was applied to a Bio-Gel A50m column (2.5 × 45 cm) equilibrated with PBS. Fractions (5 ml) were collected; the elution monitored by absorption at 280 nm and the void volume peak, containing material greater than 50 million Daltons, was collected. In the centrifugation procedure, ascites were successively centrifugated at 300 **g** for 10 min, 800 **g** for 30 min, 10 000 **g** for 30 min and 100 000 **g** for 1 h. The pellet was then washed in PBS and floatation of membrane vesicles on a discontinuous sucrose gradient was performed at 4°C as previously described (Raposo *et al*, 1996). Membrane vesicles from both procedures were then centrifuged at 100 000 **g** for 1 h at 4°C. The pelleted membrane vesicles were resuspended in PBS and the quantity of protein was determined by the Bradford microassay method (Bio-Rad Laboratories, Hercules, CA, USA), using BSA as a standard.

To compare the distributions of proteins in membrane materials isolated by these procedures, SDS–PAGE using a 8–15% acrylamide gel (Laemmli, 1970) was performed, followed by silver staining (Bio-Rad Laboratories, Hercules, CA, USA). The stained gels were analysed by Kodak 1D Image Analysis software (Eastman Kodak, Rochester, NY, USA).

### Western immunoblots analyses of ‘exosomes’ protein expression

Western immunoblotting was performed to analyse the presence of specific proteins, including HLA-A, PLAP, TSG101, FasL, B23/nucleophosmin. Proteins from each ‘exosomes’ preparation (35 *μ*g) were applied per lane of a 4–20% SDS–PAGE gel. The proteins were electrophoretically separated by the method of Laemmli (1970) and analysed by Western immunoblot as previously described (Brown *et al*, 1993), probing overnight at 4°C with either rabbit polyclonal anti-FasL (1 *μ*g ml^−1^, Calbiochem, San Diego CA, USA), rabbit polyclonal anti-PLAP (1 *μ*g ml^−1^, Abcam, Cambridge, MA, USA), mouse monoclonal anti-TSG101 (1 *μ*g ml^−1^, Abcam, Cambridge, MA, USA), rabbit polyclonal anti-B23/nucleophosmin (1 *μ*g ml^−1^, Santa Cruz Biotechnology, Santa Cruz, CA, USA), or goat polyclonal anti-HLA-A (1 *μ*g ml^−1^, Santa Cruz Biotechnology, Santa Cruz, CA, USA) as the primary antibodies and peroxidase-conjugated anti-rabbit, goat, or mouse immunoglobulin as the secondary antibody. The bound immune complexes were visualised by enhanced chemiluminescence (ECL, Amersham Life Sciences, Arlington Heights, IL, USA) and quantitated by densitometry (Un-Scan-it Software, Silk Scientific Corp., Orem, UT, USA).

### Expression of signalling proteins, TcR/CD3-*ζ* and JAK3

Jurkat E-61 cells, a human T-cell lymphoma, was obtained from the American Type Culture Collection (Manassas, VA, USA). These cells were utilised as an *in vitro* assay for lymphocyte modulation by ascites-derived ‘exosomes.’ This T-cell line was grown in RPMI 1640 medium supplemented with 0.1 mM nonessential amino acids, 1 mM sodium pyruvate, 200 mM L-glutamate, 100 *μ*g ml^−1^ streptomycin and 100 IU ml^−1^ penicillin in a humidified 5% CO_2_ chamber at 37°C. Cell viability was evaluated by trypan blue exclusion. All cultures utilized for this study were >95% viable.

For bioassay of CD3-*ζ* expression, viable Jurkat cells (10^6^ cells ml^−1^) were incubated in a medium supplemented with 400 *μ*g ml^−1^ isolated ‘exosomes’ for 48 h and were compared with unexposed Jurkat cells or Jurkat cells exposed to the analogous gradient fractions from control sera. After 2 days, the cells were centrifuged, the cell pellet washed and used for protein analysis. To assess CD3-*ζ* protein, the cell pellet was lysed using 50 mM HEPES, pH 7.2, 150 mM NaCl, 5 mM EDTA, 1 mM sodium orthovanadate, 2.5% Triton X-100, 200 *μ*g ml^−1^ trypsin/chymotrypsin inhibitor, 200 *μ*g ml^−1^ chymostatin and 2 mM PMSF. The cell lysate was assayed for protein by the BioRad protein assay (Bio-Rad Laboratories, Hercules, CA, USA). The modulation of signalling proteins was analysed by Western immunoblot using a 15% SDS–PAGE gel, as described above with mouse monoclonal anti-CD3-*ζ* and mouse anti-JAK 3 antibodies (Santa Cruz Biotechnology, Santa Cruz, CA, USA) as the primary antibodies. As an additional loading control, blots were also probed using rabbit polyclonal anti-*β*-actin (Santa Cruz Biotechnology, Santa Cruz, CA, USA).

### DNA fragmentation

The induction of apoptosis was examined as DNA fragmentation in T lymphocytes. Cultures of Jurkat cells (2 × 10^5^ cells ml^−1^) following no treatment, treatment with 400 *μ*g ml^−1^ membrane vesicles, or treatment with analogous material from control serum were removed from the flask after 24 h. DNA was isolated and electrophoresed as previously described (Gibb *et al*, 1997). After electrophoresis, DNA bands were visualised by 0.5 *μ*g ml^−1^ ethidium bromide staining.

### Isolation of ‘exosomes’-associated inhibitory components

Using a continuously eluting electrophoresis system (Bio-Rad Laboratories, Hercules, CA, USA), the components of ‘exosomes’ mediating suppression of CD3-*ζ* were analysed. ‘Exosomes’ from two cancer patients (500 *μ*g each) were applied to 12.5% acrylamide preparative column gels and each gel was run at 100 V. Fractions (1 ml) were eluted from the bottom of the gel, monitoring at 280 nm. Proteins from aliquots (200 *μ*l) of each protein-containing fraction were precipitated using a 2-D clean up kit to remove SDS (Amersham Biosciences, Arlington Heights, IL, USA). The precipitated protein was resuspended in PBS by sonication and assayed for *ζ* suppression in Jurkat cells. The fractions exhibiting *ζ* suppression were concentrated and analysed by SDS–PAGE on a 12.5% gel and subsequently by Western immunoblotting with anti-FasL antibody.

### Statistical Analysis

Western blot analyses of TSG101, HLA, PLAP, B23, FasL, CD3-*ζ*, JAK3 were performed at least twice. Densitometric quantitation of bands on each gel was standardised to a control lane included on that gel and compared by the Kruskal–Wallis test. In the remainder of experimental data, all relative absorbance determinations were performed at least twice and the mean ± standard error of the mean for each sample was calculated. Tests with *P*<0.05 were considered statistically significant. Statistical analysis was performed using InStat (GraphPad, San Diego, CA, USA).

## RESULTS

### Comparison of membrane vesicles and exosomes from ascites of ovarian cancer patients

**Figure 1:**
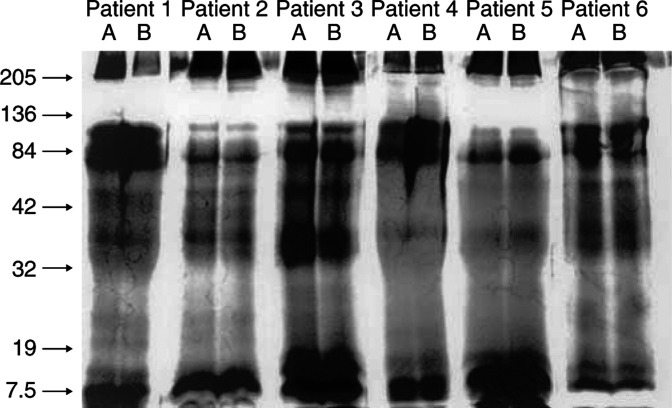
SDS–PAGE separation of chromatographically isolated membrane vesicles (designated lanes A) and centrifugally isolated ‘exosomes’ (designated lanes B) from the ascites of the same ovarian cancer patients, visualised by silver staining.

**Figure 2:**
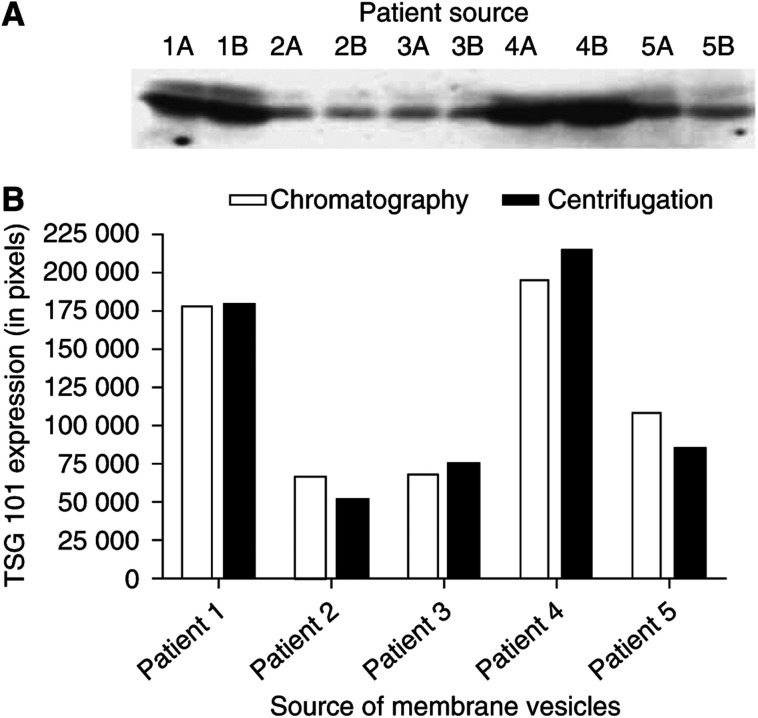
(**A**) Representative Western immunoblots of SDS–PAGE separation of chromatographically isolated membrane vesicles (designated lanes A) and centrifugally isolated ‘exosomes’ (designated lanes B) from the ascites of the same ovarian cancer patients, incubated with anti-TSG101 antibody and visualised by ECL. (**B**) Densitometric analysis of TSG101 expression on these membrane vesicle isolates.

Using ascites from ovarian cancer patients, membrane vesicles isolated by size exclusion chromatography were compared with ‘exosomes’ isolated by density gradient centrifugation. After standardising for protein content, the membrane materials were electrophoretically separated and the protein composition was compared (Figure 1). Based on general protein composition, chromatographically isolated membrane vesicles appear to be identical to centrifugally isolated ‘exosomes’ obtained from the same ascites. Subsequent analysis of TSG101 (previously demonstrated to be associated with exosomes) (Stoorvogel *et al*, 2002) on these membrane vesicles also indicated that the membrane materials isolated by both procedures produced identical results (Figure 2).

### Characterisation of antigens associated with membrane vesicles

**Figure 3:**
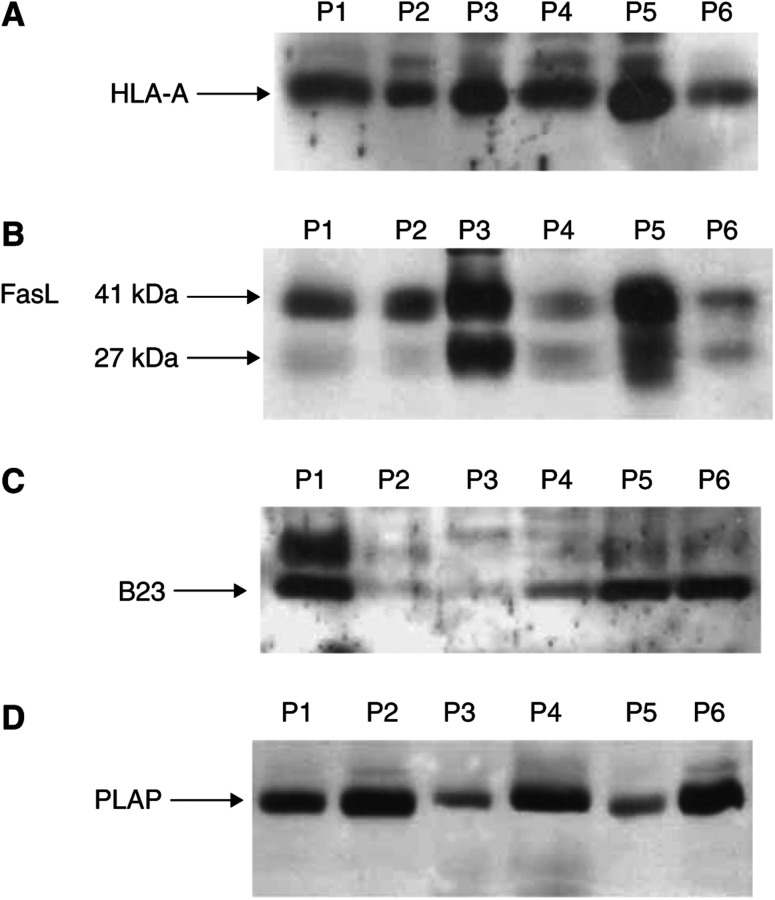
Western immunoblots demonstrating presence of HLA-A, Fas ligand, B23/nucleophosmin, and placental alkaline phosphatase on centrifugally isolated ‘exosomes’ from the ascites of ovarian cancer patients.

Western immunoblot analysis of centrifugally isolated ‘exosomes’ demonstrated the presence of class I histocompatibility antigens in the ascites-derived membrane vesicles, confirming the expression previously shown for tumour-derived ‘exosomes’ (Figure 3A). To define the origin of these membrane vesicles, the ‘exosome’-association of placental-type alkaline phosphatase (PLAP), a plasma membrane enzyme linked with ovarian cancer, was also assayed (Figure 3D). Recent analyses at the gene and protein levels have demonstrated PLAP expression in germ cell tumours and syncytiotrophoblasts and only rarely is the protein or message observed in normal adult tissues (Shigenari *et al*, 1998; Goldsmith *et al*, 2002). All ‘exosome’ isolates stained positive for PLAP, indicating the probable tumour origin of these membrane materials. Our previous work demonstrated the presence of autoantibodies bound to membrane vesicles, which were reactive with B23/nucleophosmin (Katsanis *et al*, 1998); this finding suggested a translocation of this nuclear protein to the cell surface for exposure to the immune system. ‘Exosomes’ from four out of six patients were positive for the presence of B23 by Western immunoblots (Figure 3C). Since other investigators have suggested the antitumour cellular immune response can be suppressed by the presence of Fas ligand (FasL) (Rabinowich *et al*, 1998; Whiteside, 2002), shed ‘exosomes’ were assessed for expression of FasL. All samples were positive for the intact 41 kDa form of FasL and two were also strongly positive for the 27 kDa cleaved form (Figure 3B). Although control sera failed to exhibit shed membrane vesicles, the analogous gradient fractions were analysed for the presence of HLA, FasL, B23, and PLAP and were found to be negative (data not shown).

### Suppression of TcR/CD3-*ζ* and JAK 3 protein by shed ‘exosomes’

**Figure 4:**
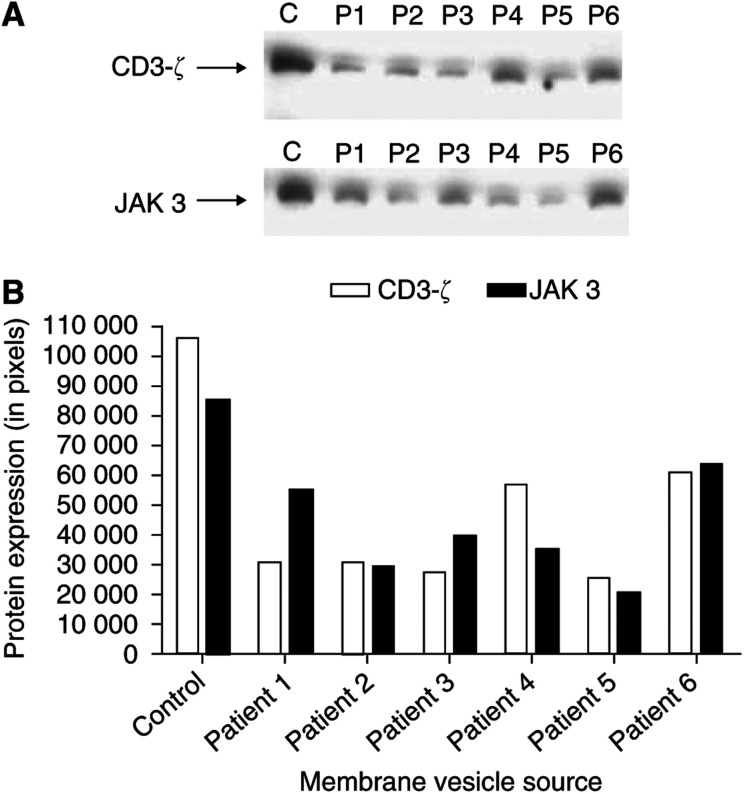
(**A**) Western immunoblots indicating the expression of CD3-*ζ* protein and JAK 3 by Jurkat cells, following incubation with 400 *μ*g ml^−1^ of the centrifugally isolated ‘exosomes’ or analogous gradient material (Control, C) for 48 h. (**B**) Densitometric quantitation of CD3-*ζ* and JAK 3 expression by treated Jurkat cells.

T lymphocytes from ovarian cancer patients have been demonstrated to exhibit a loss of CD3-*ζ* expression and enhanced apoptosis (Rabinowich *et al*, 1998; Whiteside, 2002). Our work has suggested that JAK 3 may represent a common link between loss of CD3-*ζ* protein and induction of apoptosis. Jurkat cells were incubated for 2 days in medium containing 400 *μ*g ml^−1^ of ‘exosomes’ or the analogous gradient fraction from control sera, and the expressions of CD3-*ζ* protein and JAK 3 were determined by Western immunoblot. As shown in Figure 4, CD3-*ζ* and JAK 3 expressions were decreased in Jurkat cells incubated with ‘exosomes,’ compared to those incubated with analogous control material.

### ‘Exosome’-mediated induction of apoptosis

**Figure 5:**
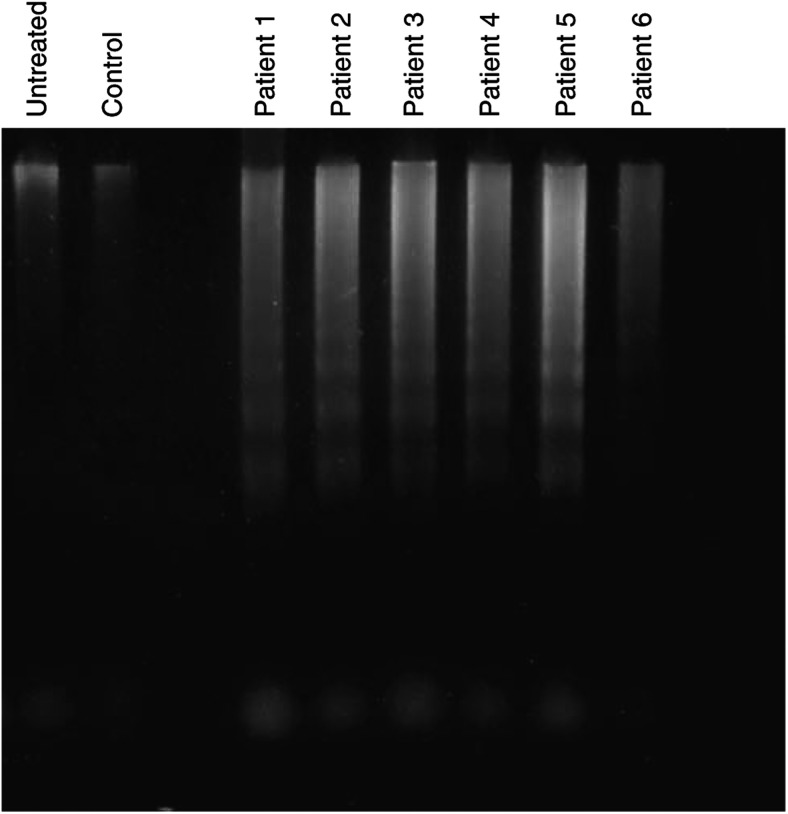
Induction of apoptosis in Jurkat T cells by 400 *μ*g ml^−1^ of the centrifugally isolated ‘exosomes’ *vs* the analogous fraction from control female controls for 24 h, as defined by DNA fragmentation.

Since it has been hypothesised that induction of T-cell apoptosis by FasL is linked to the loss or decrease of TcR/CD3-*ζ* expression (Rabinowich *et al*, 1998), the capability of FasL-expressing ‘exosomes’ to induce apoptosis was examined by assessing the induction of DNA fragmentation (Figure 5). ‘Exosomes’ isolated from ovarian cancer patients’ ascites induced significant apoptosis compared to untreated Jurkat cells or Jurkat cells incubated with the analogous control fraction.

### ‘Exosome’-associated inhibitory components

**Figure 6:**
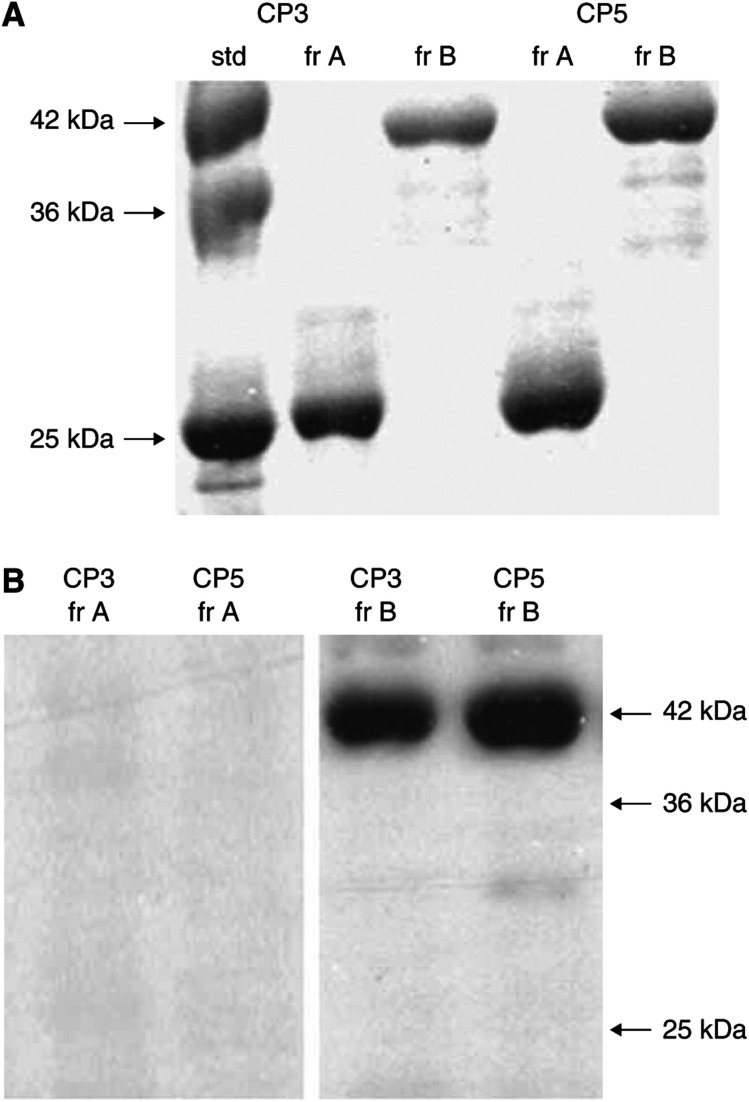
Identification of the ‘exosomes’-associated inhibitors of CD3-*ζ* expression. ‘Exosomes’ were fractionated by continuously eluting electrophoresis and fractions were subsequently assayed for *ζ* suppression in a Jurkat bioassay. The fractions suppressing *ζ* expression were examined by SDS–PAGE with silver staining (**A**). Western immunoblotting of the two fractions with anti-FasL demonstrated reactivity with the 42 kDa component, but not with the 26 kDa (**B**).

Since the preparations of ‘exosomes’ were observed to suppress CD3-*ζ* and JAK 3 and to induce apoptosis within Jurkat cells, the characteristics of the inhibitory component were evaluated. ‘Exosomes’ from two patients were electrophoretically separated by continuously eluting electrophoresis. For each of the ‘exosome’ preparations, two separate fractions were identified. When these components were visualised by silver staining on standard SDS–PAGE gels, one component appeared at 26 kDa and the second at 42 kDa (Figure 6A). A separate preparation of this material was analysed by Western immunoblotting with anti-FasL antibody, since it has been implicated in suppression of CD3-*ζ*, induction of T-cell apoptosis and exhibits molecular weight forms at these molecular weights. Only the 41 kDa component stained positive for FasL (Figure 6B). Since our previous studies indicated that the anti-FasL antibody used recognized both forms, the absence of reactivity with the 26 kDa components indicates that it is not FasL.

## DISCUSSION

The shedding of membrane vesicles by tumour cells and their subsequent appearance in blood specimens and malignant effusions (ascites and pleural fluids) of cancer patients has been recognised for over 25 years. These shed membrane vesicles have been implicated in the immunosuppressive events associated with advanced cancers. Owing to the presence of immunogenic tumour antigens, early evidence suggested a role in the loss of surface antigens by tumours and in competition for antibody binding (Taylor *et al*, 1984, 1989; Katsanis *et al*, 1998; Gerçel-Taylor *et al*, 2001). Later, data demonstrated that as tumours progressed and specific tumour subpopulations were selected or developed, these shed membrane vesicles acquired the ability to suppress cellular immunity (Taylor and Black, 1985, 1986; Pelton *et al*, 1991; Gerçel-Taylor *et al*, 2002; Taylor *et al*, 2003). The acquisition of this suppressive activity by membrane vesicles parallels the development of anergy within these cancer patients.

In the 1980s, the release of ‘exosomes’ by lymphoid cells was observed (Pan *et al*, 1985; Johnstone *et al*, 1987; Théry *et al*, 2002; Stoorvogel *et al*, 2002). These ‘exosomes’ appeared to play a central role in communication between lymphocytes and dendritic cells, mediating the development of cellular responses or suppressing excessive activation, as in AICD. The phenomena of shedding ‘exosomes’ have also been demonstrated with other haematopoietic cells, such as mast cells and platelets, and it is thought to play important roles mediating their actions (Raposo *et al*, 1997). Recent studies have isolated ‘exosomes’ from tumour cells *in vivo* and *in vitro* and have demonstrated the presence of tumour-associated antigens and class I MHC antigens (Wolfers *et al*, 2001; André *et al*, 2002a, 2002b; Chaput *et al*, 2004). These studies have suggested that these tumour-derived ‘exosomes’ can serve as direct vaccines or can be processed by autologous dendritic cells, which can then be used to stimulate the anti-tumour response.

Our findings, as well as those of other investigators, have demonstrated that membrane vesicles isolated from ovarian cancer patients’ peripheral blood and malignant effusions appear to be identical, in terms of general protein composition and presence of specific marker proteins (Taylor *et al* 1980, 2002, 2003; Graves *et al*, 2004). However, to date, it is unclear whether the shed tumour-derived membrane vesicles are the same or distinct from tumour-derived ‘exosomes.’ Using our published chromatographic procedure for membrane vesicle isolation and the published density gradient centrifugation protocol for ‘exosomes’ purification, shed membrane materials were isolated from ascites of ovarian cancer patients. Based on protein staining, there are no significant differences between the composition of these two preparations (Figure 1). Further analysis of a marker, TSG101, previously demonstrated to be associated with ‘exosomes,’ also demonstrated no significant differences between chromatographically and centrifugally isolated membrane materials (Figure 2).

Since ‘exosomes’ have been shown to exhibit HLA antigens and tumour-associated antigens, the presence of specific antigens was confirmed on ascites-derived ‘exosomes.’ These ‘exosome’ isolates expressed HLA-A antigens at similar levels (Figure 3A), as well as expressing two tumour-associated antigens (PLAP and B23) (Taylor *et al*, 1984; Gerçel-Taylor *et al*, 2001) that have previously been demonstrated to be present on membrane vesicles isolated from both peripheral blood and ascites (Taylor *et al*, 1980, 2002, 2003) and to generate humoral responses in autologous patients (Figure 3C and D). We further demonstrated that these shed tumour-derived ‘exosomes’ expressed primarily the proapoptotic, intact 41 kDa FasL (Figure 3B).

Whiteside (2002) has proposed a link between T-cell apoptosis and decreased CD3-*ζ* expression. Her work previously demonstrated that coincubation of T lymphocytes with FasL-expressing ovarian tumour cells resulted in both loss of CD3-*ζ* and induction of lymphocyte apoptosis (Rabinowich *et al*, 1998). The expression and release of FasL also appears to play a critical role of AICD of peripheral T cells and the nonlymphoid FasL expression contributes to peripheral lymphocyte deletion via apoptosis (Green *et al*, 2003). This study addressed the consequences of ‘exosome’ expression of biologically active molecules, such as FasL. When ascites-derived ‘exosomes’ were incubated with T cells (Jurkat cells) for 48 h, suppression of both CD3-*ζ* and JAK 3 proteins were observed (Figure 4). This suppression was observed using 400 *μ*g ml^−1^ of membrane proteins. Our previous work demonstrated that this membrane material was present at a level of 2.06±0.73 mg ml^−1^ in advanced ovarian cancer patients. These FasL-containing ‘exosomes’ were further analysed for their ability to induce apoptosis (Figure 5). Induction of significant DNA fragmentation was observed in T cells (Jurkat cells) within 24 h. Fractionation of ‘exosomes’ revealed two distinct fractions mediating suppression of CD3-*ζ* (Figure 6A): 26 and 42 kDa. Western immunoblotting demonstrated the 42 kDa component to be FasL, while the 26 kDa component did not react with anti-FasL. Current studies are focused on sequencing the 26 kDa component.

Our analyses of tumour-derived ‘exosomes’ have confirmed their identity with shed membrane vesicles; however, the use of the term ‘exosomes’ has caused a failure to link them with their published immunologic consequences. Since shed tumour membrane vesicles or ‘exosomes’ do possess tumour-derived antigens that can elicit autologous immunologic responses, the potential use of these proteins as targets for vaccines is promising; however, these antigenic components need to be separated from those components mediating suppression of T-cell signalling and inducing apoptosis. Based on their known immunosuppressive characteristics, their potential use intact as vaccines should be performed with caution.
